# Supplementary material for: Pan-genome insights into type VI secretion systems and their functional repertoires in Enterobacter
Source: mSystems. 2026 Apr 16;11(5):e01781-25. doi: 10.1128/msystems.01781-25 (PMC13185608; doi:10.1128/msystems.01781-25)
Supplement: Supplemental material — Figures S1 to S7; captions for Tables S1 to S4. [file msystems.01781-25-s0001.docx]

**Supplementary figure 1**

**
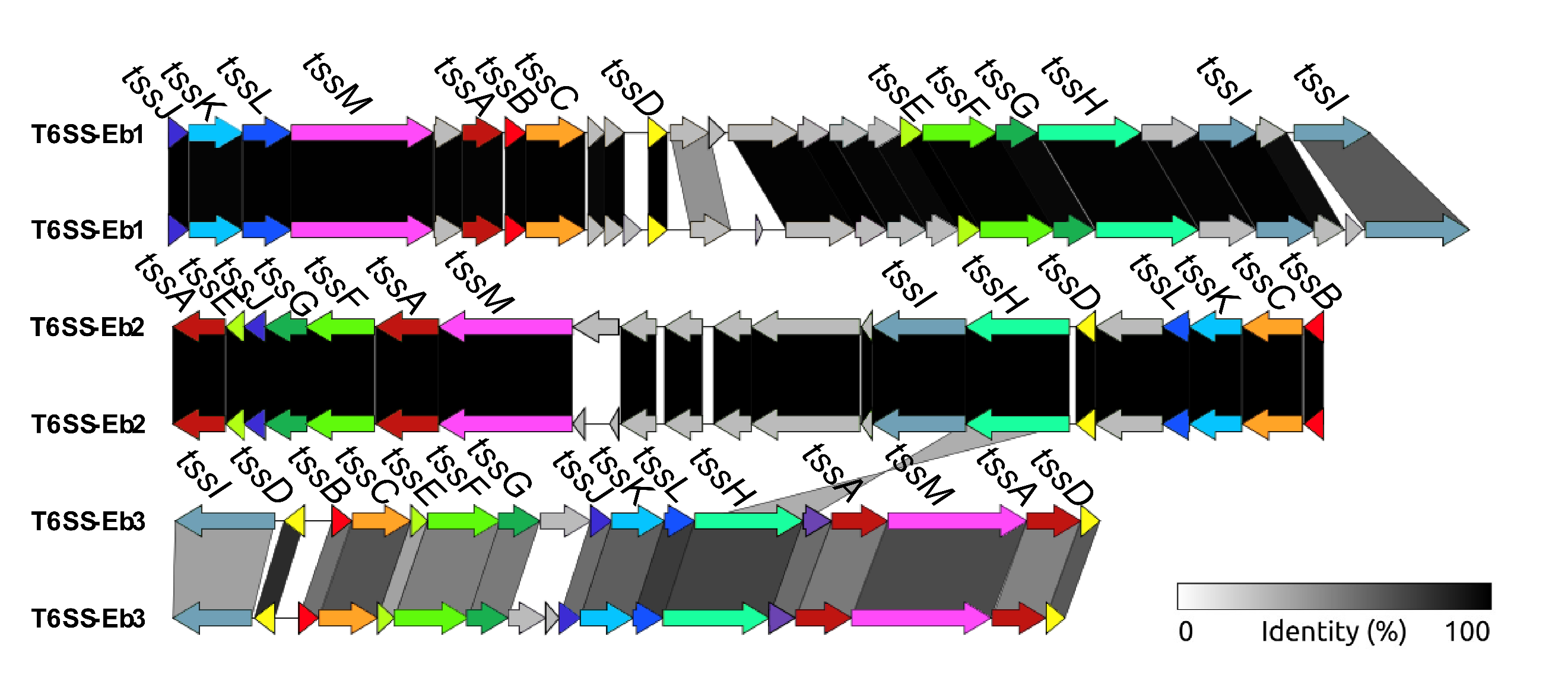
**

**Supplementary Figure 1** | **The three types of T6SS loci were compared both within and between groups.** Colored regions represent core components, while grey regions indicate other genes. Shading between genes denotes sequence identity, with the color intensity reflecting amino acid sequence identity. Genes without connecting lines indicate no detectable identity.

**Supplementary figure 2**


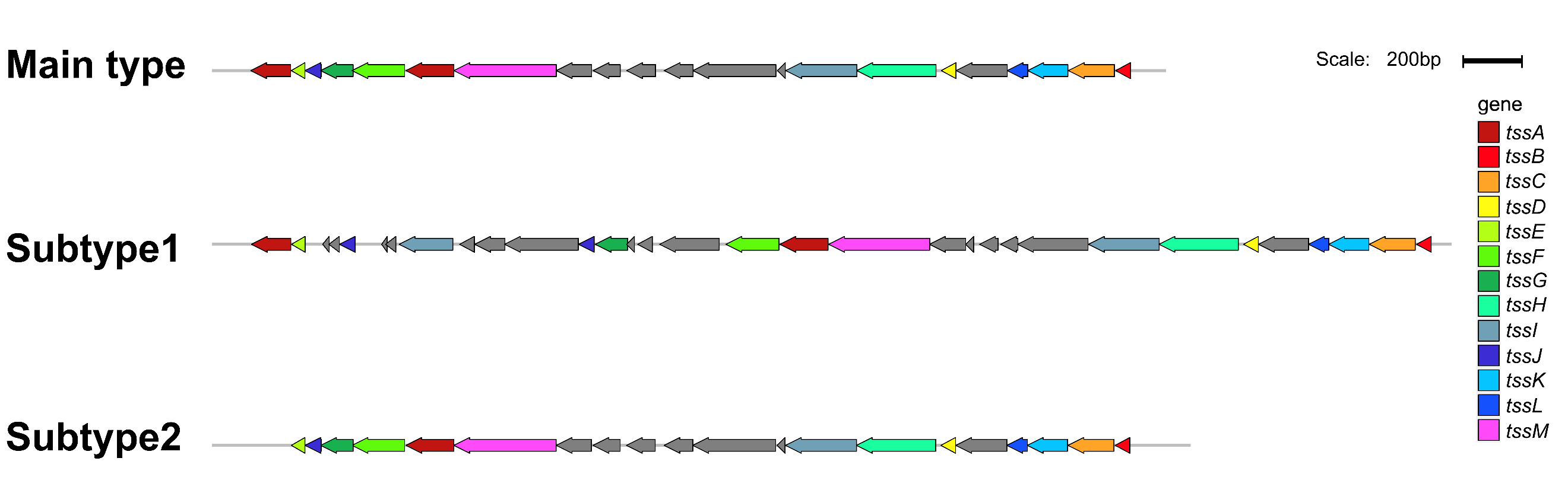


**Supplementary Figure 2** | **Main type and two subtypes of T6SS-Eb2.** Genes scale in length and *tssA*-*tssM* colored by different components.

**Supplementary figure 3**


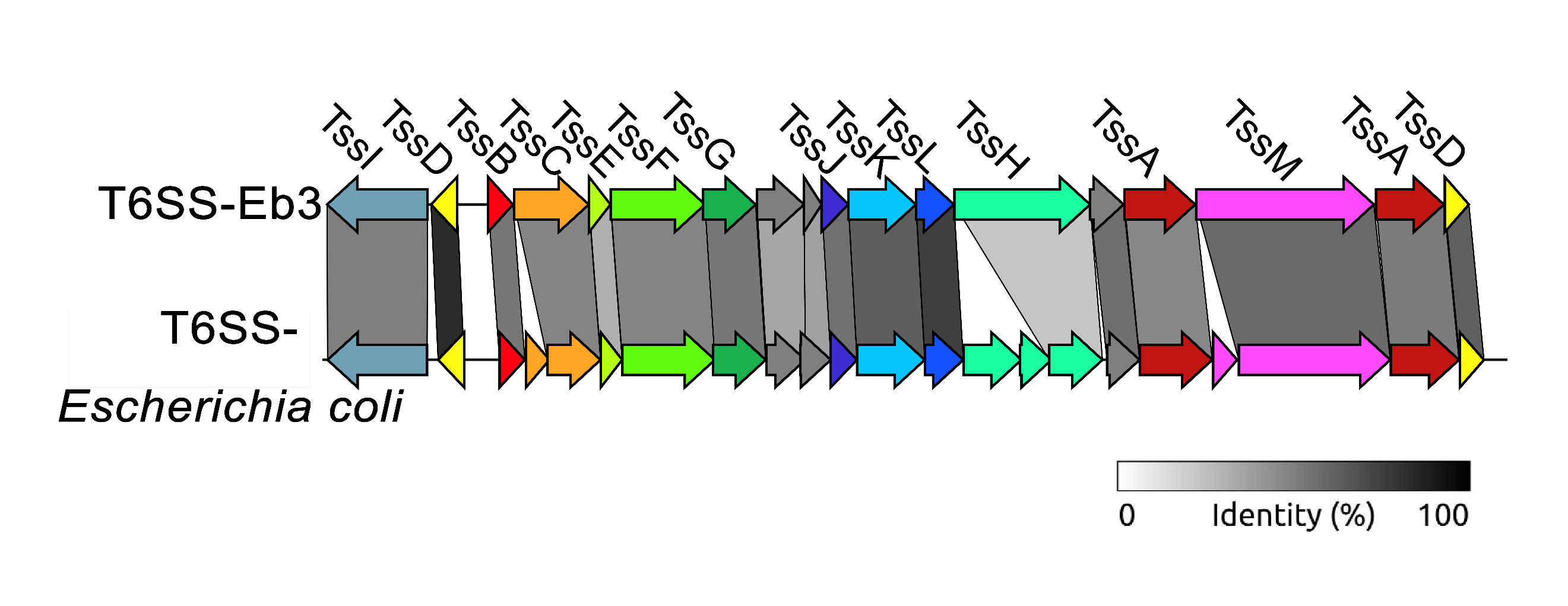


**Supplementary Figure 3** | **T6SS-Eb3 compared with T6SS in *E. coli* K1.** Colored regions represent core components, while grey regions indicate other genes. Shading between genes denotes sequence identity.

**Supplementary figure 4**


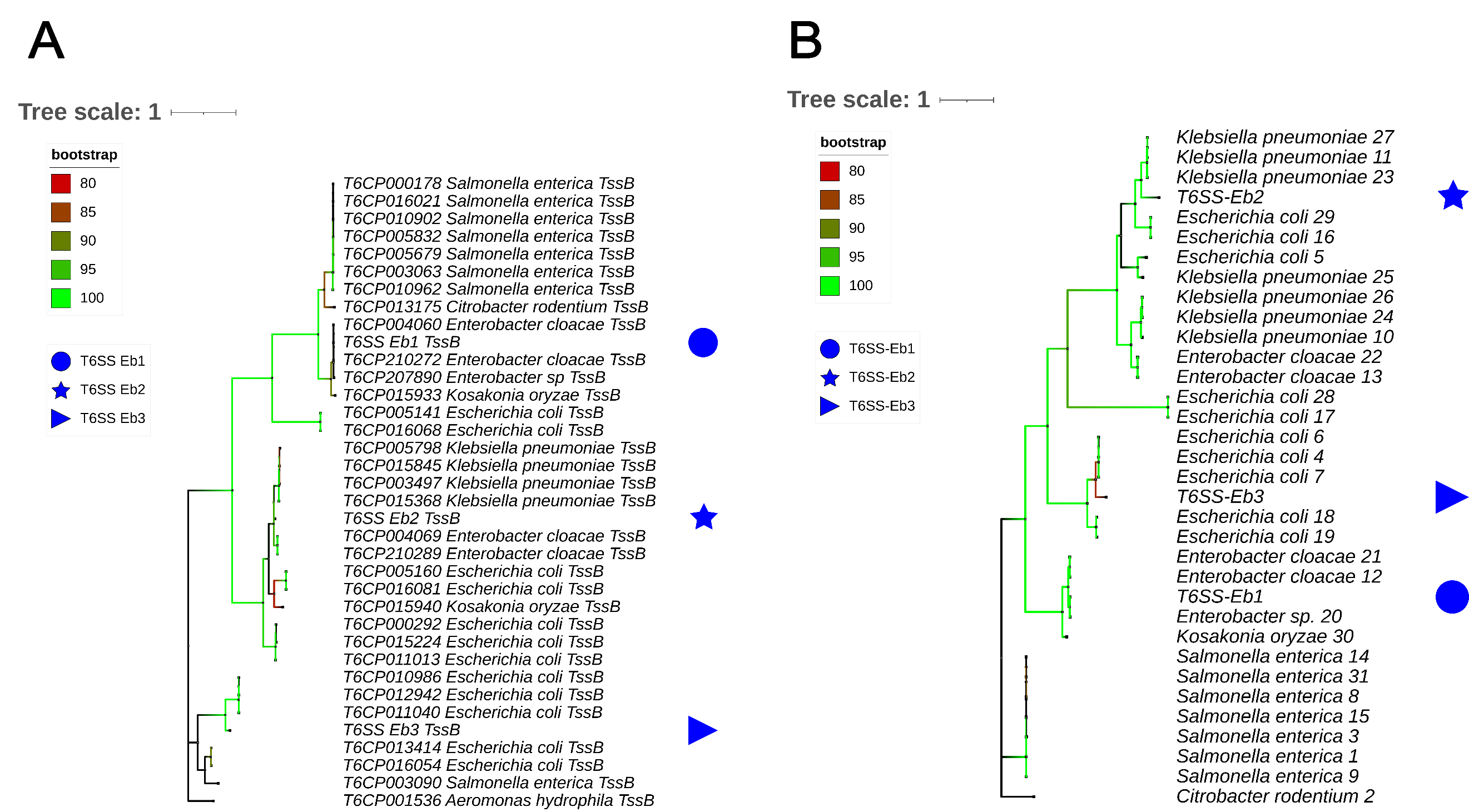


**Supplementary Figure 4 | Phylogenetic relationships of the three T6SSs and *Enterobacteriaceae* family.** (A) Single-gene phylogenetic tree inferred from TssB protein sequences. (B) Multi-locus phylogenetic tree constructed using concatenated conserved T6SS core components. In both panels, T6SS-Eb1, T6SS-Eb2, and T6SS-Eb3 are highlighted by a blue circle, star, and triangle, respectively.

**Supplementary figure 5**


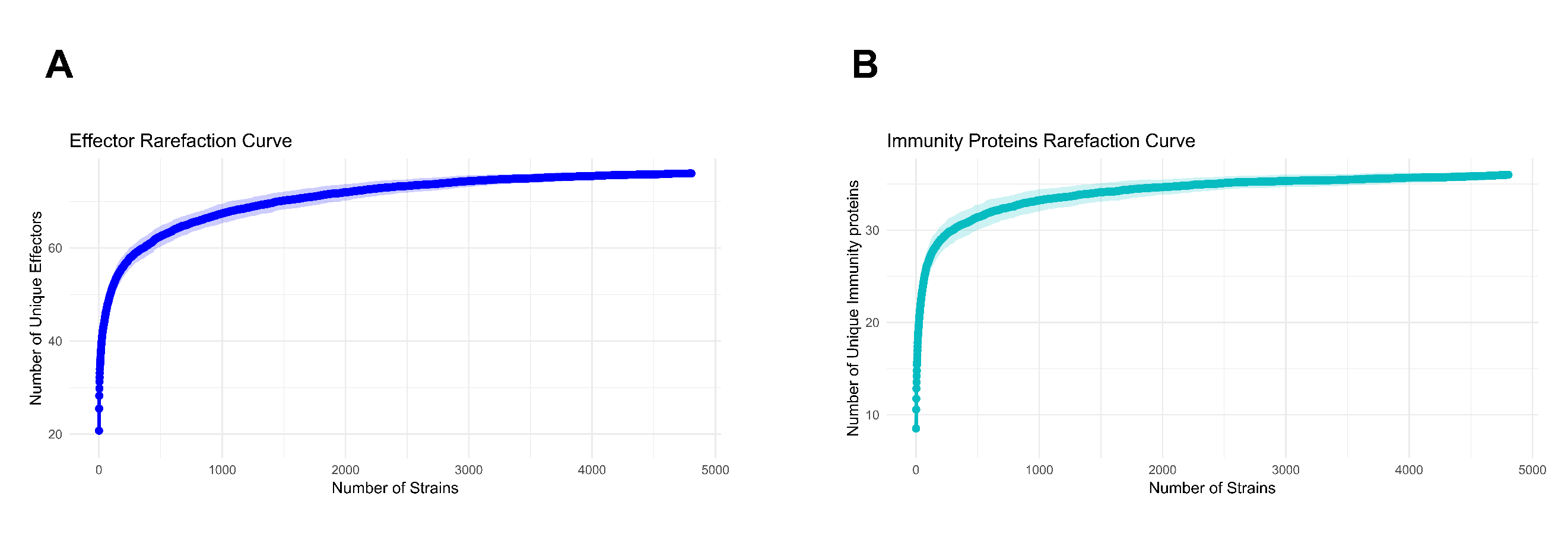


**Supplementary Figure 5** | **The rarefaction curves showing observed effectors(A) and immunity proteins(B) richness in *Enterobacter* spp..**

**Supplementary figure 6**


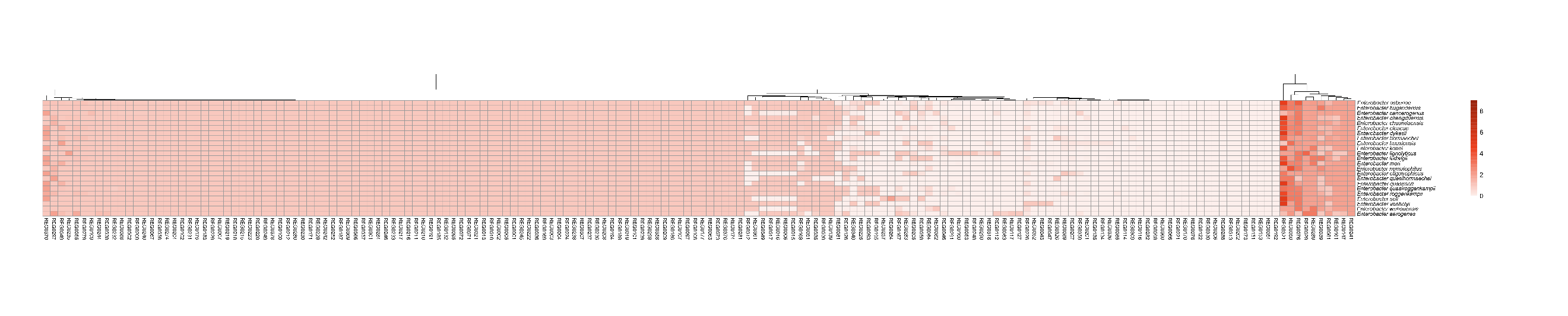


**Supplementary Figure 6** |**The heatmap displays the abundance and category of regulators across species, with color intensity representing abundance.** Scale means the number of homologous genes.

**Supplementary figure 7**





**Supplementary Figure 7** | **Similar with Fig. 4 the number of T6SS-associated proteins are not significantly higher in strains with intact T6SS-Eb2 than in strains without a T6SS-Eb2 all the time.** (**A, C,** and **E**) The histogram plots given the differences in effector(**A**), immunity protein(**C**), and regulator(**E**) in *E. hormaechei* were used as a representative to analyze whether the presence of T6SS-Eb2 correlates with the abundance of related proteins. The X-axis shows the average number of these proteins in genomes with (red) and without (blue) T6SS-Eb2. A statistically significant difference was observed between the two groups (****P*-value < 0.001), as determined by a nonpaired Student’s *t*-test. (**B, D,** and **F**) The bar graphs represent the distribution in *E. hormaechei* of effector(**B**), immunity protein(**D**), and regulator(**F**), with the x-axis showing the number of these proteins and the y-axis showing the logarithmic number of genomes. (**G, I,** and **K**) The histogram plots given the differences in effector(**G**), immunity protein(**I**), and regulator(**K**) in *E. cloacae* were used as a representative to analyze whether the presence of T6SS-Eb2 correlates with the abundance of related proteins. The X-axis shows the average number of these proteins in genomes with (purple) and without (grey) T6SS-Eb2. A statistically significant difference was observed between the two groups (****P*-value < 0.001, 0.01<**P*-value<0.05) and no statistically significant difference (*P*-value > 0.05), as determined by a nonpaired Student’s *t*-test. (**H, J,** and **L**) The Bar graphs represent the distribution in *E. cloacae* of effector(**H**), immunity protein(**J**), and regulator(**L**), with the x-axis showing the number of effectors and the y-axis showing the logarithmic number of genomes.

**Supplementary Table 1** | **Taxonomic assignment of genomes based on GTDB-Tk analysis.**

**Supplementary Table 2** | **Pairwise associations of T6SS loci in *Enterobacter*.**

**Supplementary Table 3** | **BLAST alignment of T6SS-Eb3-containing contigs against the NCBI non-redundant database.**

**Supplementary** **Table** **4** | **Annotation of T6SS-Eb1−associated and T6SS-Eb2−associated orthologous groups in *Enterobacter.*** This table lists orthologous protein clusters significantly enriched in *Enterobacter* genomes with complete T6SS-Eb1 and T6SS-Eb2 (log₂ P/A > 2.4, χ² *P*-value< 0.05).
